# Cultural evolution in the laboratory: evolution of cooperative altruistic punishing

**DOI:** 10.1017/ehs.2025.10018

**Published:** 2025-09-15

**Authors:** William M. Baum, Peter J. Richerson

**Affiliations:** Department of Environmental Science and Policy, University of California, Davis, CA, USA

**Keywords:** Altruism, cooperation, cultural evolution, micro-society, public-goods game, punishment

## Abstract

Culture consists of practices – behaviour patterns – shared by members of a group. Some attempts to demonstrate evolution of cultural practices in the laboratory have shown evolution of material products, such as paper aeroplanes. Some attempts have shown evolution of actual group behaviour. The present experiments demonstrated evolution of group coordination across generations in punishing defection in a public-goods game. Cost of punishing defection varied across replicates that consisted of series of groups (generations) of 10 undergraduates each. Each generation played the game anonymously for 10 rounds and could write messages to the other participants and punish defection every round. The effectiveness of punishment depended on the number of participants choosing to punish. In Experiment 1, cultural transmission from generation to generation consisted of written advice from one generation read aloud to the next generation. In Experiment 2, transmission from generation to generation consisted of having some participants return from the previous group. The cost of punishing varied across replicates: zero, one, two or five cents. In both experiments, the evolution of altruistic punishing was strongly dependent on the cost of punishing. The results add to plausibility of studying evolution of complex behaviour patterns like cooperation in the laboratory.

## Graphical abstract

**Figure:**
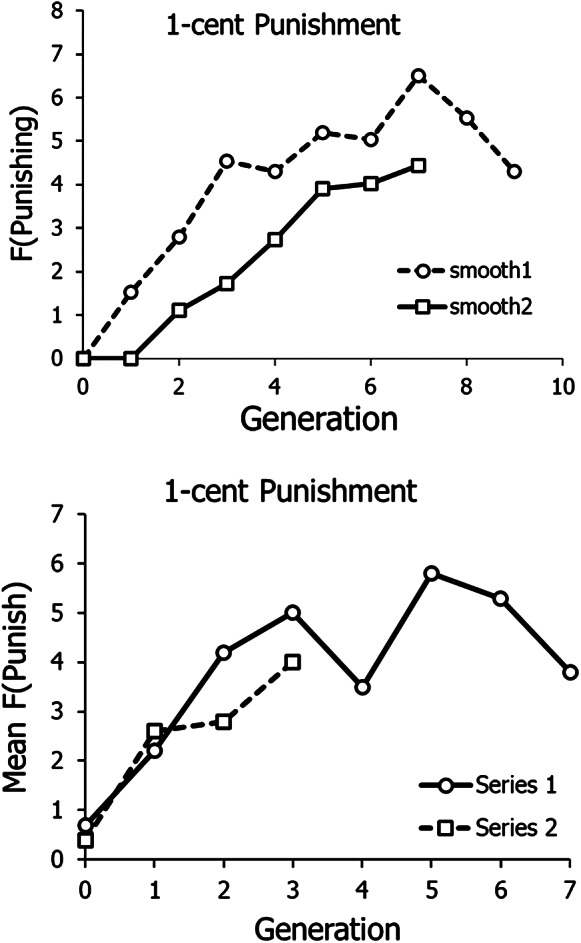

## Social media summary

Two experiments showed that cultural evolution of a social policy can be simulated in the laboratory.

## 1. Introduction

A group’s culture consists of the practices common among the group’s members – whether building boats, processing acorns, giving rules, enacting ceremonies or enforcing norms – that are activities, behaviour. Some practices may be understood in relation to individuals’ welfare, for example, all who process acorns follow a certain method. Other practices may only be understood in relation to the welfare of the group as a whole, for example, forbidding marriage between close kin prevents inbreeding. This second category may be called a culture’s *policies*. Change in a cultural policy requires cooperation of multiple members of the group; one person alone rarely can effect a change of policy.

Cultural evolution is change in a group’s practices across generations that results from processes of innovation and selection. It has been studied using mathematical models based on the recursion equation formalism used to study organic evolution, with appropriate modifications to account for the important differences between genetic and cultural transmission and evolution (Baum, 2017; Boyd & Richerson, 1985; Cavalli-Sforza & Feldman, 1981). Empirically, real-world instances of cultural change are studied by historians and historically minded social scientists (Newson & Richerson, 2021; Rogers, 1995; Wasserman & Cullen, 2016) and by implementing experimental models in the laboratory (Carey, 2009; Baum et al., 2004; Caldwell & Millen, 2008; Insko et al., 1983; Kempe & Mesoudi, 2014).

Early experimental models attempted to show cumulative cultural evolution of material products: paper aeroplanes and raw-spaghetti towers (Caldwell & Millen, 2008, 2009). The general procedure is to define a group as a generation and to allow each generation to inform the next. For example, in the experiment with paper aeroplanes, each group of three participants worked for 2.5 minutes on an aeroplane, after which one participant in the group was replaced with a naïve person iteratively for several generations (Caldwell & Millen, 2008).

Experiments with material products face the problem of how to measure cumulative cultural change. Most experiments assessed cumulative culture by effectiveness: how far a paper aeroplane flew; height of a tower; and maximum weight borne by a bridge or basket. An experiment by Kempe and Mesoudi (2014) measured progress more directly by allowing groups of three 12 minutes each to solve a jigsaw puzzle from the beginning after being allowed to see what either one group or three groups before them had done and measuring evolution by the number of pieces correctly placed. The researchers observed evolution across four generations when groups viewed three earlier attempts. Improving manufacturing of an object, however, indicates only the result of behavioural innovations in construction, not the innovations themselves. Measuring the change in behaviour presents an additional challenge. After all, the manufacture of material objects is behaviour, and cumulative evolution of manufacturing – introducing innovations – is evolution of behaviour.

An alternative to studying cumulative evolution of material objects is to study evolution of a policy, which is group behaviour. A number of experiments have examined cumulative evolution of group behaviour outside the laboratory (e.g. Miu et al., 2018). Several recent summaries of the field exist (Boyd, 2018; Laland, 2017; Mesoudi, 2011; Newson & Richerson, 2021).

**Figure 1.**
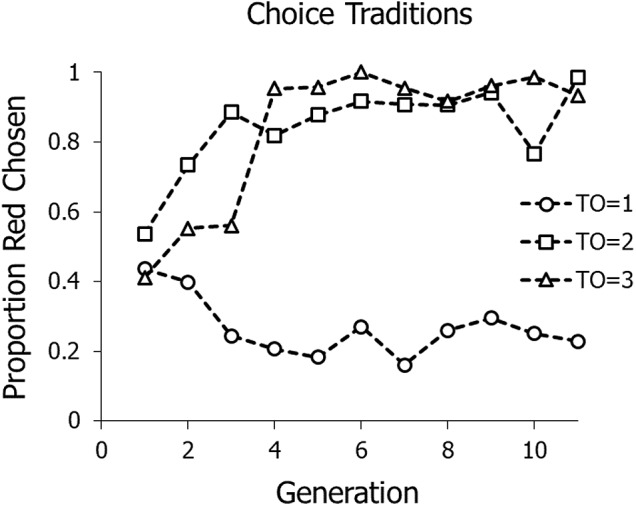
Evolution of policy in Baum et al. (2004). Participants chose between solving red anagrams earning 10 cents and blue anagrams earning 25 cents with various timeout (TO) durations following choice of a blue anagram. Timeouts were 1, 2 and 3 minutes across conditions.

Baum et al. (2004) studied cumulative evolution of policy rather than material objects. They allowed each group of four to work at a task: solving anagrams. The group chose by consensus an anagram from either of two stacks, printed on blue cards or on red cards. If they chose a red card, after solving the anagram they each received 10 cents and immediately chose another card. If they chose a blue card, after solving the anagram they received 25 cents but then had to wait through a timeout before they could solve another anagram. Each generation worked for 12 minutes, after which a naïve participant replaced the oldest group member. Across three conditions, the duration of the timeout following a blue anagram was either 1, 2 or 3 minutes. When the timeout was 1 minute, they could earn more money by choosing blue; when the timeout was 3 minutes, they could earn more money by choosing red; and when the timeout was 2 minutes, the two paid off equally. Variation in the time taken to solve the anagrams obscured these differences. When the new person joined, the other three participants immediately began instructing and persuading the newcomer as to how to choose. In each condition, a policy of choosing the better alternative evolved across generations. Figure 1 summarizes group choice across generations. When the timeout after 25 cents was 1 minute, a policy of choosing blue evolved. When the timeout was 3 minutes, a policy of choosing red evolved. When the timeout was 2 minutes, a policy of choosing red evolved – the immediate reward dominated when choice made no difference.

Although the results shown in Fig. 1 represent evolution of a relatively simple policy – only two choice alternatives and choice by consensus – the experiment’s success suggests that more complex policies might allow experimental models. One type of challenge groups commonly face is evolving policy for managing common resources, such as irrigation water available to all but potentially overexploited by some members. A common method for studying group policy with respect to a common resource is the public-goods game (e.g. Fehr & Gӓchter, 2002; Ostrom et al., 1994, 1992). A large body of research has examined many different aspects and implications of public-goods games and other economic games (Camerer, 2011). In each round of the game, participants may contribute to a public good, usually a common fund. The total contributed to the public fund is then multiplied by a constant greater than 1.0 (e.g. doubled), and then distributed equally among participants. The game presents a dilemma, because anyone who does not contribute – who ‘free rides’ – gains more than everyone else, but if no one contributes, no one earns anything. To prevent free-riding, the group may employ tools such as messaging and punishment. Baum et al. (2012) found that groups of five playing a public-goods game substantially increased contributions if they were allowed to write messages that were read aloud after every round, even though the messages were anonymous and unrelated to any sanctions – what economists refer to as ‘cheap talk’. Contributions also increase when participants have the ability to punish those who contribute too little (defectors; e.g. Gürerk et al., 2006). The ability of group members to punish other members who break rules or norms may be crucial to ensuring cooperation. In a public-goods game, as in natural resource sharing, groups can ensure cooperation in the form of contributions to the public good (Pedrazzini et al., 2025).

Chaudhuri et al. (2006) pursued the possibility of studying evolution of cooperation in groups across generations. Groups of five played a public-goods game for 10 rounds and then left written advice for the next group to follow. They found evolution of cooperation in the form of increased contributions to the public good across three generations when advice was ‘common knowledge’ – that is, read aloud to the group. Hillis and Lubell (2015) extended the results of Chaudhuri et al. by showing that when each group leaves common-knowledge advice for the next group, the advice grows to advocate more for contributing.

### 1.1. Cooperative punishing

A policy entails rules and norms that require group members to cooperate. Punishment for non-cooperation may be crucial for evolution of cooperation (Gürerk et al., 2006). A social group may generally punish failures to cooperate with relatively mild punishers like disapproval. To be effective, mild punishment must be frequent and consistent. If only one person disapproves openly of misbehaviour, or only a few, the misbehaviour likely will continue. To prevent misbehaviour, such punishment must be cooperative – group members must coordinate their punishing. In a public-goods game, where cooperation is beneficial for everyone, free-riding is misbehaving. We arranged a punishment regime to reflect this contingency: to be effective, punishing had to be cooperative. No one participant’s punishing was costly enough to free riders to deter them.

The present Experiment 1 adapted the procedure of Chaudhuri et al. (2006) to studying evolution of cooperative altruistic punishment in groups of 10 participants. Earlier pilot experiments showed that groups of five contributed at a high level and rarely punished, because most groups contained no defector. We found that groups of 10 nearly always include some defectors, and we wanted to study cooperative punishment in that more challenging situation.

In the present experiments, the groups could write messages (common knowledge) and could punish defectors every round. To punish low contributions, a participant set a criterial contribution, and any contribution less than the criterion resulted in a small fine (10 cents). The total fine increased additively according to the number of participants who chose to punish, so punishing defections sufficiently to deter free-riding required participants to cooperate. On each round, each participant could contribute up to 50 cents to the public fund, and the total was doubled and distributed equally. If everyone contributed 50 cents (total of 500 cents), everyone netted 50 cents. If nine participants contributed 50 cents and one person contributed nothing (total of 450 cents), the defector netted 90 cents while everyone else netted 40 cents. If four people chose to punish, the defector netted only 50 cents, the same as if everyone had contributed 50 cents. To render defection fruitless, at least four people had to choose to punish. Thus, too few participants choosing to punish would still allow defection to be profitable, and effective deterrence of free-riding required at least four participants to punish cooperatively.

This punishment regime modelled enforcement of a rule or norm, for example, when members express disapproval on witnessing someone transgressing (or defecting from cooperating). In addition, communication each round led to some ability to advocate for punishment and negotiate punishment coordination. Even in modern societies with professionalized justice systems, citizens act individually. They can report a crime or not, act as witnesses or not, lobby police and prosecutors, vote on taxes to support the justice system, and take direct action against perpetrators.

Punishing in the present experiments was altruistic when participants had to pay to punish. They had to sacrifice their own personal welfare for the sake of the group if punishing was costly. The net cost or benefit to an individual of their decision to punish depended on the effect of their punishment on the other players’ behaviour. If sufficient individuals punished, and their punishment motivated defectors to cooperate, their collective benefits could exceed their costs. Nevertheless, canny defectors achieved the highest payoffs, always by a small margin, by contributing nothing when rates of punishing were low, and what they saved by not investing in the public good exceeded the cost any punishment received. This agrees with theoretical models in which between-group selection, not implemented in our experiment, is required to sustain altruistic punishment in the long run (e.g. Gürerk et al., 2006). Within groups the slight advantage of strategic free riders may eventually erode cooperation, whereas groups that maintain high rates of cooperation will differentially proliferate at the expense of those that produce low amounts of the public good (Boyd et al., 2003).

These experiments aimed to create a viable model of cultural evolution in the laboratory. Compared with culture in the wild, which occurs in large populations and provides many models to emulate, laboratory studies must aim to capture basic processes without being too simplistic to be informative or too logistically challenging. If they are successful, they may offer a limited way to gain insight into cultural evolution, in the same way that evolution in *Drosophila* and *Escherichia coli* may shed light on evolution of slower-breeding species like humans. Experiments with social dilemmas typically provide groups with either communication or punishment to influence cooperation. The present experiments included both. In Experiment 1, each generation left written advice, all of which was read aloud to the new generation. Experiment 2 resembled Experiment 1 in most respects, including messaging and punishment, but introduced an alternative method of intergeneration transmission to advice: bringing back experienced participants (‘elders’) to allow generations to overlap.

Conducting these experiments and presenting the results, we relied on replication of multigeneration lineages to show the reliability and repeatability of the results. We omitted null-hypothesis significance testing (NHST) because such inferential statistics tell us nothing about repeatability. Many publications have pointed this out, although their explanations often still go unheeded (e.g. Gigerenzer, 2004; Goodman, 2008; Haller & Krauss, 2002).

The experiments aimed to: (a) assess whether cultural evolution of a complex policy, cooperative rule-enforcing, might be modelled in the laboratory; (b) study the effect of cost of punishing; (c) compare different modes of intergenerational transmission; and (d) assess the efficacy of within-generation communication. The cost of punishing varied across conditions: zero, 1, 2 or 5 cents.

## 2. Experiment 1: transmission by advice

### 2.1. Method

#### Participants

A total of 720 undergraduate students at University of California, Davis, recruited from the Psychology Department’s paid-subject pool, served. Of these, 237 appeared to be male, and 483 appeared to be female. All were between 18 and 21 years old. The Institutional Review Board at UC Davis approved the protocol.

#### Apparatus

Each group of participants sat around a table (153 cm wide by 229 cm long) in a windowless room. This was large enough that 10 people could sit around it, but close to one another, intended to provide a sense of belonging to a group. To prevent neighbours from observing each other’s work, 10 cardboard boxes (28.5 cm high, 44 cm wide and 23.5 cm deep) were arranged around the edges of the table such that each participant sat in front of a box. The height of the box allowed each participant a clear view of the faces of all the other participants of their experimental generation, also intended to generate a sense of belonging to a group. Participant messages often included joking, flirting and incidental chit-chat, indicating that they were socially aware of others in the group. Each box contained a pen, a printed copy of the instructions, a folder with a recording sheet inside and a six-page booklet enclosed in a folder (the ‘communication folder’), with nine spaces for writing comments and a full page for writing advice. The page for advice was marked off into three sections, labelled ‘contributions’, ‘punishment’ and ‘messages’. The participant wrote inside the box, thus preserving privacy and anonymity. On a wall at the front of the room, a white board allowed summary outcomes to be written for all participants to see. A computer on a desk in a corner allowed researcher assistants to record data directly into a workbook. The recording sheet consisted of a grid with 10 rows – one for each round of the game – and columns for writing one’s contribution, to indicate whether one chose to punish, and three columns for feedback from the experimenters (share, amount subtracted due to punishment, and private account balance). The instructions and recording sheet appear in Appendix A.

#### Procedure

Sessions were conducted at the same time weekly from April 2009 to June 2017 as long as the university was in session. When 10 participants had arrived, each received a consent form to sign. After they gave consent, the first author (W.M.B.) read out the instructions, telling the participants to read their copies along with the out-loud instructions. He solicited questions answered with quotes from the instructions. All generations except the Series-1 progenitor groups received advice from the previous 10-participant generation, which W.M.B. read aloud. In Series 1 of 1-cent and 5-cent punishment, the progenitor generation received no advice, but to see whether strong advice might make a difference to the course of evolution, the first generation in Series 2 of 1-cent and 5-cent punishment received strong advice (i.e. 9 of 10 members advising to punish) taken from Generation 11 of the free-punishment series. The first round of the game then began. Each experimental session lasted approximately 1.5 h.

The participants played 10 rounds of a public-goods game in which the group’s contribution was doubled and then distributed equally among participants. All play was silent and anonymous. Each participant began with an endowment of 500 cents in their private account. On each round, each participant wrote a contribution from zero to 50 cents on the recording sheet and indicated whether to punish or not. Choosing to punish meant subtracting the cost, if any, from the participant’s private account and specifying a criterial contribution that would result in a fine of 10 cents for anyone and everyone who contributed less than the criterial contribution. Participants routinely set this criterion at 50; it was an uninformative feature of the game. Punishment increased additively and linearly – that is, the more people punished, the larger the potential fine: if two people punished, the fine could be 20 cents, and so on. If four people punished, and three people defected, all three defectors received a fine of 40 cents. The recording sheets included columns for the cost of punishment, subtracting the cost, and the criterion (from 1 to 50). The last three columns, filled in by the experimenters, gave feedback. (See recording sheet in Appendix A.)

The recording sheets (inside folders) were collected and the choices recorded. The total contribution, double the contribution, the share per person and the number of people who chose to punish were called out and written on the white board. Then participants were told they could write a message for the round. After messages were written, the communication folders were collected and W.M.B. read all messages aloud, providing what Chaudhuri et al. (2006) call ‘common knowledge’. Prior research showed that such within-generation comments, even if ‘cheap talk’, substantially increased contributions (Baum et al., 2012). Participants could not identify which of the other nine was the author of a given message. The recording sheets were handed back, and the next round began. At the end of 10 rounds, participants wrote advice for the next group and each participant privately received their earnings plus an additional $5.00 for showing up.

Five series of groups were conducted. Each group was a generation, and each series of groups constituted a lineage and a replicate. The cost of punishment varied across series, but within each series, the cost of punishment remained fixed. The costs implemented were zero (one series), 1 cent (2 series) and 5 cents (2 series). The number of generations (i.e. groups) within a series was extended until one could see whether the frequency of punishment was approximately stable. Every message was coded afterward according to whether it included: (1) urging to contribute, (2) chat, (3) praise, (4) urging to punish or not, (5) threat and (6) disapproval.

## 3. Results

**Figure 2.**
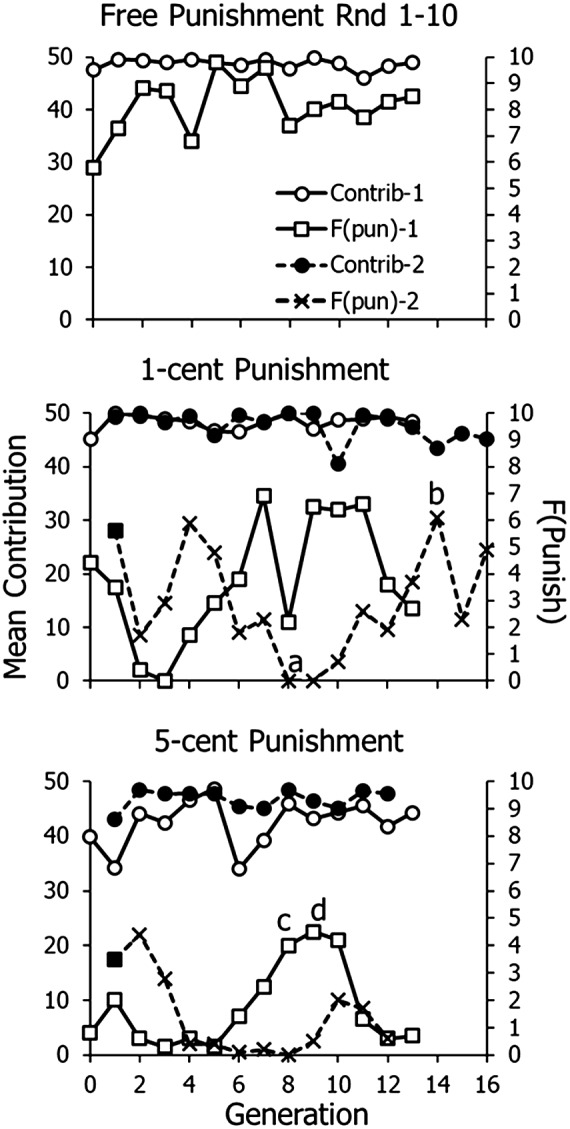
Group behaviour across generations in Experiment 1, replicates 1 and 2. Mean contribution in cents is plotted on the left-hand vertical axis, and number of participants choosing to punish is plotted on the right-hand vertical axis. At point *a*, two super-cooperative generations occurred in a row. At points *b, c* and *d*, advice to punish was unusually strong. Filled squares indicate initial generations that received strong advice to punish. Circles represent contributions. Squares and X’s represent number punishing.

Figure 2 shows means across Rounds 1–10 of contributions and numbers of participants choosing to punish plotted against generations. The top graph (‘free’ punishment; zero cost) shows that when cost of punishing was minimal, frequency of choosing to punish (F(pun); right-hand vertical axis) reached high levels. Contributions were high as well, but contributions tended to be high throughout the experiment. Apart from the increase in punishing from the progenitor (Generation zero; no advice), to Generation 1, no evolution of punishing appears for free punishment because the frequency of punishing is uniformly high.

The middle graph in Fig. 2 shows frequency of punishing for the two 1-cent series. In Series 1, punishing initially fell, reaching zero at Generation 4. After that, punishing increased and stabilized between three and seven participants choosing punishment per generation. The mean across the last seven generations was 5.0 participants choosing to punish. The slope of a regression line fitted to Generations 5–13 was close to zero (0.04). To see whether initial bias to punish would affect results, whereas Series 1 began with no advice, Series 2 began with advice taken from Generation 11 of free punishment with 9 of 10 advocating for punishment (filled square). Series 2 still replicated the same sort of pattern as Series 1, although Series 2 took more generations to fall all the way to zero. At the point marked *a*, two generations in a row were perfectly cooperative, contributing the maximum and never punishing. After that, punishing evolved. It reached a maximum at the point marked *b*, when punishing ramped up to eight people choosing by Round 5. The mean across the last four generations equals 4.25. The slope of a regression line fitted to Generations 13–16 equalled zero.

The bottom graph in Fig. 2 shows the results when the cost of punishing was 5 cents. In Series 1, punishing remained at a low level until Generation 5, after which it rose to about 4.0 for three generations. In the groups marked *c* and *d*, defections (low contributions) drove more players to punish, which resulted in rising contributions, and in Generations 8–11, seven to nine participants advised to punish. After that, punishing fell back to a low level. Series 2 began with strong advice to punish (filled square), as in Series 2 of 1-cent punishment, but punishing fell to a low level and remained at a low level, replicating the results of Series 1.

**Figure 3.**
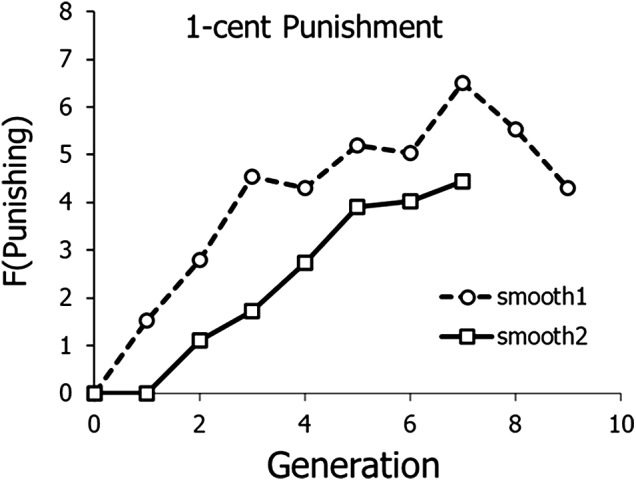
Frequency of punishing in Experiment 1 with 1-cent cost of punishing. Data are from middle panel of Figure. 2 and are smoothed according to a three-point running average.

Figure 3 shows frequency of punishing across generations in the two 1-cent punishment series. The data from Fig. 2 were smoothed with a three-point running average. Because both lineages dropped to zero punishing before punishing evolved, the graphs begin with the generation in which frequency of punishment fell to zero: Generation 4 in Series 1; Generation 9 in Series 2. Both graphs show the evolution of punishment across generations.

## 4. Experiment 1 discussion

Altruistic punishing evolved when the cost of punishing was 1 cent – that is, low but not free (Figs 2 and 3). Series 2 replicated the results of Series 1, both in the initial decline and the subsequent evolution. When punishing was free, frequency of punishing was high and relatively invariant (Fig. 2, top), as it tends to be in many, but not all, societies cross-culturally (Herrmann et al., 2008). When punishing cost 5 cents, frequency of punishing was low and showed no clear evolution, because punishing tended to fall off in the final generations (Fig. 2, bottom).

The shortcomings of the methods of Experiment 1 include: (a) the absence of any cost of punishing between 1 and 5 cents; and (b) the apparent lack of clear analogy between the division of generations and actual cultural evolution, where generations overlap. Instead of formal advice, one generation (e.g. parents, teachers, elders) usually influences the behaviour of the next generation by example and informal instruction. Experiment 2 aimed to address these shortcomings.

## 5. Experiment 2: overlapping generations

This experiment resembled Experiment 1 in most respects, but included a condition with 2-cent cost of punishing and substituted overlap in generations instead of read-out advice. Participants were invited to return and participate again in subsequent generations. As these returnees had experience with the game, they corresponded to ‘elders’, and their messages were identified as coming from people who had participated before.

### 5.1. Method

#### Participants

A total of 830 undergraduate students at University of California, Davis, recruited from the Psychology department’s paid-subject pool, served. Of these, 225 appeared to be male and 605 appeared to be female. All were between 18 and 21 years old. The Institutional Review Board at UC Davis approved the protocol.

#### Apparatus

The experimental set-up was the same as in Experiment 1, except the instructions differed, and the communication folder included similar spaces for the 10 rounds, omitted any space for advice to the next generation, and included a box to check if the participant had participated before. When reading out messages, W.M.B. announced if the participant had participated before. The instructions appear in Appendix B. The recording sheet was the same as in Appendix A.

#### Procedure

The game, the method of instructing, recording, punishing, messaging and payment were all the same as in Experiment 1, except that no advice was written or read out, and writing messages preceded each round instead of following the round. After W.M.B. had read out instructions and answered questions, the first round began with the invitation to write messages. All messages were read aloud. At the end of the 10 rounds, the experimenter announced that all participants could return and that they could participate up to three times. Returnees self-selected. They only needed to show up before the session began. Participants were paid privately as in Experiment 1.

Sessions were conducted at the same time weekly from November 2015 to February 2020, before the COVID-19 pandemic, and from November 2022 to November 2023, after the pandemic, as long as the university was in session. Six series of generations (replicates) were conducted: one with 5-cent cost of punishing, three with 2-cent cost of punishing and two with 1-cent cost of punishing. As in Experiment 1, to see if an initial bias toward punishing would affect results, Series 2 of 1-cent punishment began with two generations of free punishment, intended to elevate frequency of punishing. Series 1 of 2-cent punishment lasted only six generations due to the end of the academic year. Series 2 and 3 of 2-cent punishment ended with two generations of free punishment to see if frequency of punishing would increase. The experiment was cut off when the COVID-19 pandemic required everyone on campus to wear masks, which would have introduced uncontrolled variation if the face-to-face structure of the experiment was important. The second 1-cent series was conducted after the pandemic, but terminated after just seven generations, because so few students were coming to campus that recruiting a group of 10 proved almost impossible. Those generations were sometimes separated by more than a week.

The number of participants (elders) who returned varied from one to five, except for three groups in which six returned. The mean number of returnees in the 1-cent punishment series was 4.0 in Series 1 and 2.9 in Series 2. In the 2-cent punishment series, the means were 4.2, 3.5 and 3.6 in Series 1, 2and 3, respectively. In the 5-cent punishment series, the mean was 3.4.

## 6. Results

### 6.1. Contributions

**Figure 4.**
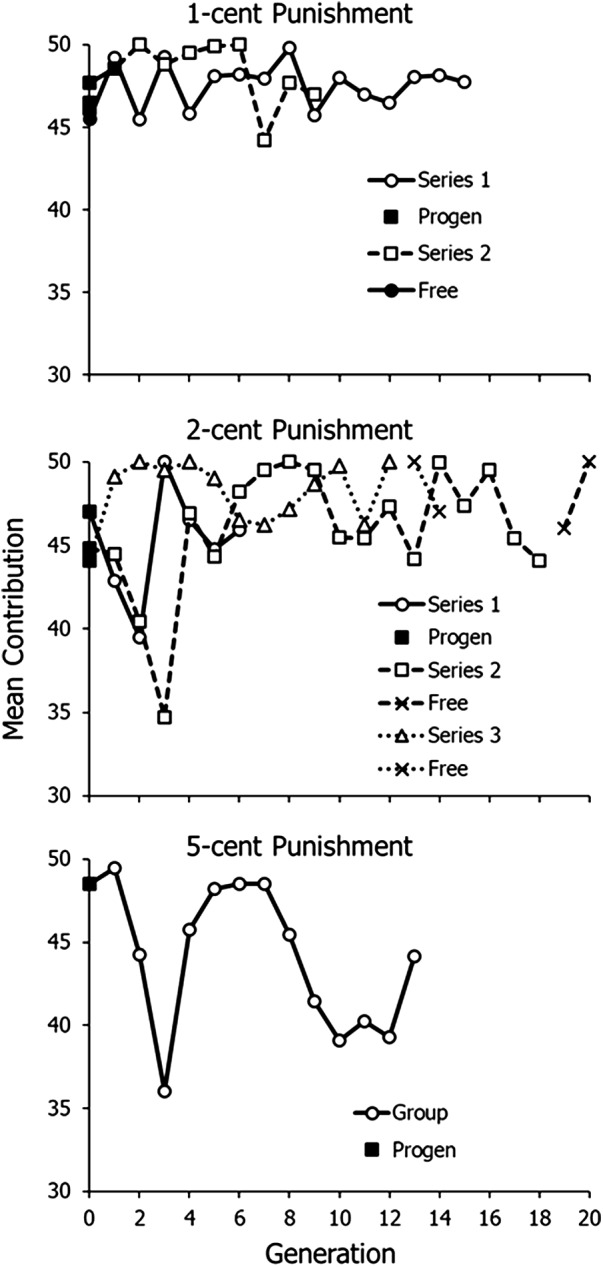
Mean contribution in cents across generations in Experiment 2. Filled squares represent progenitor generations. Solid lines represent Series 1. Dashed lines represent Series 2. Dotted lines represent Series 3. Filled circles and X’s represent generations in which punishment was free.

Figure 4 shows the mean contribution across the 10 rounds plotted by generation. The filled squares, labelled ‘progen’ for progenitor, show contributions in groups with 10 naïve participants. These generally contributed relatively generously, averaging to around 45 cents. When cost of punishing was 1 cent, contributions remained high, but only rarely averaged to 50 cents. With 2-cent punishment, contributions initially fell, but then increased after three or four generations and remained relatively high. Subsequent free punishment (X’s) had little effect on contributions. With 5-cent punishment, contributions fell to a relatively low level after nine generations.

### 6.2. Punishment

**Figure 5.**
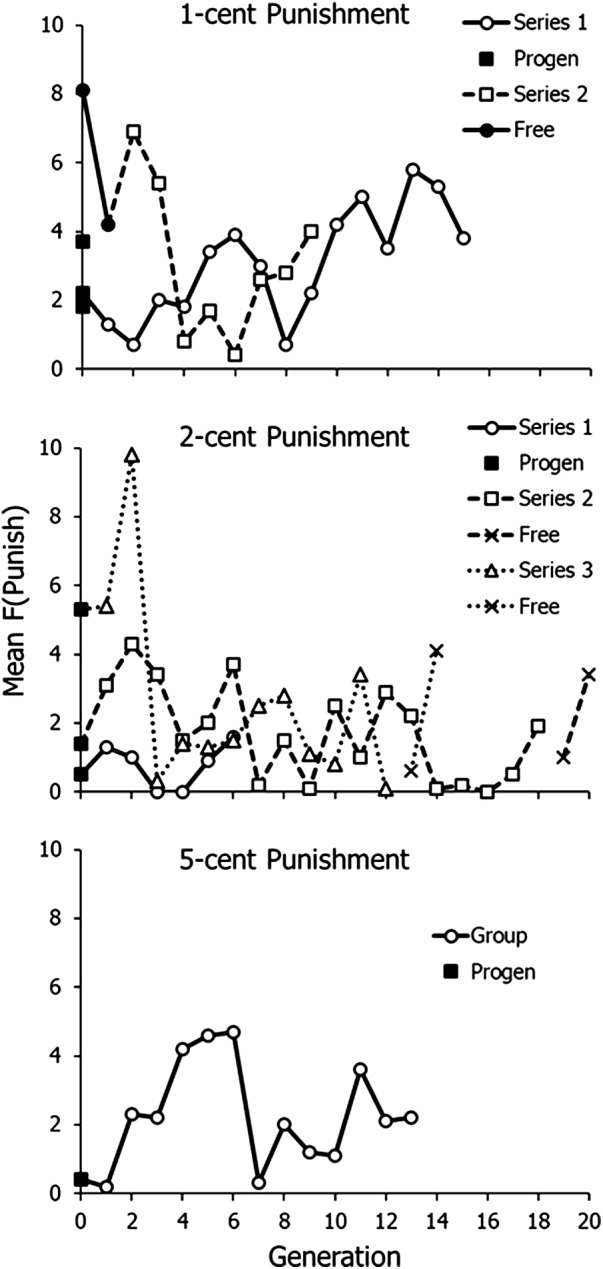
Mean number of participants choosing to punish across generations in Experiment 2. Symbols and lines as in Fig. 4.

Figure 5 shows mean frequency of choosing to punish, ‘F(punish)’, across the 10 rounds plotted by generation. The meaning of symbols is the same as in Figure 4. Progenitor groups (filled squares) chose to punish at low frequencies, except for the progenitor in the third 2-cent punishment series. The pattern of initial decline followed by evolution was replicated. In Series 1 of 1-cent punishment, frequency fell to a low level after eight generations and then levelled off from Generation 10 onwards. The slope of a regression line fitted to Generations 10–15 was close to zero (0.03), and the mean across Generations 10–15 equalled 4.6. In the second series of 1-cent punishment, which began with a high frequency of punishing from the two free-punishment groups (filled circles, solid line), punishing fell to a low level at Generation 6 and then increased from there onwards.

When punishment cost 2 cents (middle graph), punishing fell to a low level and never increased beyond about 2–3 people punishing, but when punishment was free in the last two generations (X’s), punishing remained low in the first of these, which included five elders who neither punished nor advocated punishing, but jumped in the second generation. When punishment cost 5 cents (bottom graph), punishing increased for several generations, but then decreased to a low level, averaging around two people punishing.

**Figure 6.**
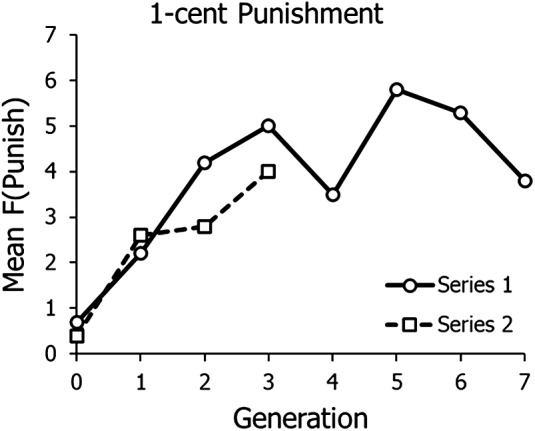
Frequency of punishing across generations in Experiment 2 when punishment cost was 1 cent. Graphs begin with the generation with the lowest level of punishing in Fig. 5 (top).

Figure 6 shows graphs of the frequencies shown in Fig. 5 (top), 1-cent cost of punishing, beginning with the generation with the lowest frequency of punishing. In Series 1, punishing evolved and then levelled off at 4–5 people punishing. The mean of the last six generations is 4.6. Series 2, which was cut off by lack of participants after the COVID-19 pandemic, showed evolution across the last four generations, and the final frequency of punishing was 4 exactly.

## 7. Experiment 2 discussion

Experiment 2, with overlapping generations, replicated the results of Experiment 1, in which written advice played the role of transmission between generations. Figure 4 shows that contributions tended to be high with 1- and 2-cent punishment, but lower when punishing cost 5 cents. Figures 5 and 6 show that punishing only evolved clearly when punishing cost 1 cent – low, but not free – as in Experiment 1. Both methods of transmission across generations seemed to work to allow evolution of punishing across generations. Both experiments – all four replicates of 1-cent punishment – showed the same pattern of initial decline followed by evolution across generations.

### 7.1. Advice and messages

**Figure 7.**
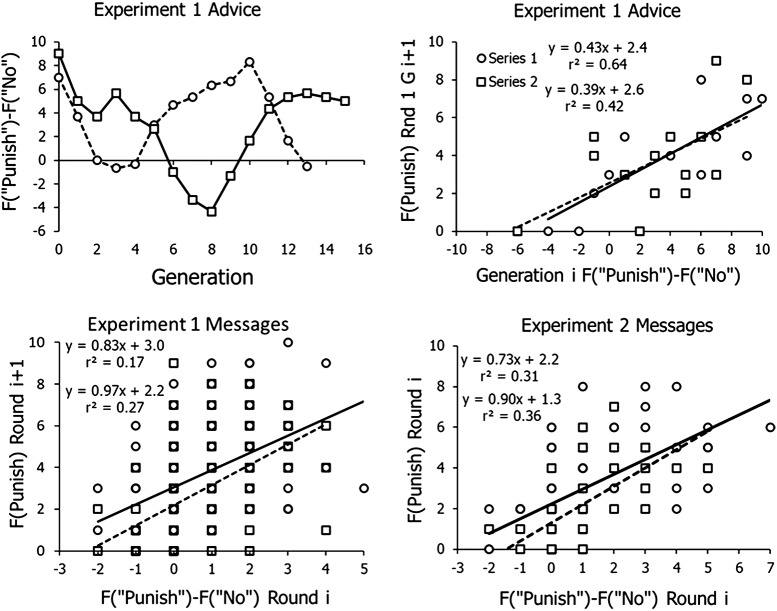
Evolution of advice and efficacy of advice and messages to punish and not to punish in the 1-cent punishment conditions. *Top left*: evolution of net advice to punish across generations. *Top right*: effect of advice on frequency of punishing, F(punish), in Round 1 of the next generation. Equations of the regression lines are shown along with variance accounted for (*r*^2^). *Bottom left*: effect of intragenerational messages in one round on punishing in the next round in Experiment 1. *Bottom right*: effect of intragenerational messages in Experiment 2.

Frequency of advice urging to contribute remained high throughout both experiments, as earlier experiments found (Baum et al., 2012). This is not surprising, because both contributors and defectors benefit from increasing contributions. Frequency of advice about punishing in Experiment 1 and intragenerational messages in both experiments played roles in the resulting evolution of punishing. Figure 7 shows their effects in the 1-cent punishment conditions. Advice about punishing was more complex than advice about contributing, because some advice urged for punishing and some urged against punishing. Cooperators benefit from others punishing but defectors benefit from low rates of punishment. To capture this contradiction, we subtracted the advice not to punish from the advice to punish, thereby creating an index that may be called ‘net advice to punish’. The top left panel shows the net advice to punish across generations. Just as punishing decreased over several initial generations, so advice to punish also decreased. When it hit a minimum, advice then evolved along with the punishing itself. The results were mixed, however. In Series 2, advice increased and levelled off, as might be expected. In Series 1, advice rose, but then decreased again even though punishing itself was frequent (Fig. 3).

The top right panel of Fig. 7 shows the efficacy of advice. The net advice to punish in a generation (*i*) is plotted on the horizontal axis, and the frequency of choosing to punish in the first round of the next generation (*i + 1*), before the participants had any experience with the game, is plotted on the vertical axis. The equations of the two regression lines appear at the upper left along with the variance accounted for (*r*^2^). The positive slopes indicate that positive net advice to punish boosted frequency of punishing in Round 1 – the advice had some effect. The positive intercepts indicate that, even when advice not to punish balanced advice to punish, still on average about two people would choose to punish.

The lower left panel of Fig. 7 shows the efficacy of intragenerational messages in the 1-cent punishment condition of Experiment 1. Frequency of punishing in a round is plotted against net advice at the end of the preceding round. Although the relations are relatively weak (*r*^2^ equal to 0.17 and 0.27), the slopes of the two regression lines are positive, indicating that advocacy of punishing had some effect. The positive intercepts indicate that two to three participants would punish on average even when advice was equally to punish or not to punish.

The bottom right panel of Fig. 7 shows the efficacy of intragenerational messages in the 1-cent punishment condition of Experiment 2. The graph shows frequency of punishing as a function of messages at the beginning of the round. The regression lines again reveal some positive effect of advice to punish reflected in the positive slopes. The positive intercepts again show some tendency to punish beyond the advice.

A question about efficacy in Experiment 2 is whether elders’ messages actually affected newcomers’ choices. Only one elder advocating punishing had no effect – that is, resulted in the same low frequency of punishing as if no one advocated punishing (0.14). If two elders advocated punishing, however, the frequency of punishing among newcomers rose from a mean of 0.14 to a mean of 0.38, indicating that the messages of the elders had some effect.

## 8. General discussion

These experiments demonstrate cultural evolution of complex policy with micro-societies in the laboratory. The earlier experiment with two-alternative choice demonstrated evolution of a consensus policy across generations (Fig. 1; Baum et al., 2004). The present experiments considered a more complex situation, asking whether coordination of policy with cost – altruistic punishment – could evolve across generations. When cost of punishing was low (1 cent), requiring a sacrifice and challenging for the participants, punishment evolved, whereas when punishing was free, punishing occurred at high levels, and when cost was higher – 2 cents or 5 cents – frequency of punishing fell to low levels and did not clearly evolve. When punishing did evolve in the 1-cent punishment conditions, frequency of punishing first declined to a low level before increasing in all four lineages. Possibly the decrease established the need for punishing to deter defections.

The present experiments add to the demonstrations of cumulative culture in the laboratory. Whereas most earlier experiments focused on manufacture of material objects, the present experiments focused on cumulating public policy. Whether studying manufacturing or public policy, these experiments aim to see if a group-level practice can evolve across generations in the laboratory. Whether studying the manufacture of baskets, the solving of a jigsaw puzzle, or altruistic punishment, the research addresses the plausibility of cultural evolution in general and in the laboratory in particular (Caldwell & Millen, 2008, 2009; Chaudhuri et al., 2006; Kempe & Mesoudi, 2014).

These experiments raise a question. What if a single group were to work at the task for the equivalent duration? For example, what if in the 1-cent punishment condition a single group of 10 participated for 15 sessions, would the results be the same? This is like asking, ‘If people could live 1000 years, would culture still evolve?’ These experiments show that culture evolves even though the players are constantly changing. Possibly culture evolves in part even *because* of the constant churn in the population; the churn is actually necessary. We have no answer for this.

Were the groups’ performances in the present experiments adaptive? To try to answer this question, we examined behaviour in the last three generations of each condition. The only rational contributions were 50 cents or zero, because any intermediate contribution could result in both the expense of contributing and fines too, and the great majority of contributions were either 50 or zero. For example, in Experiment 1, the number of contributions greater than zero but less than 50 equalled 14 and 6 (out of 300) in Series 1 and 2 of 1-cent punishment.

**Figure 8.**
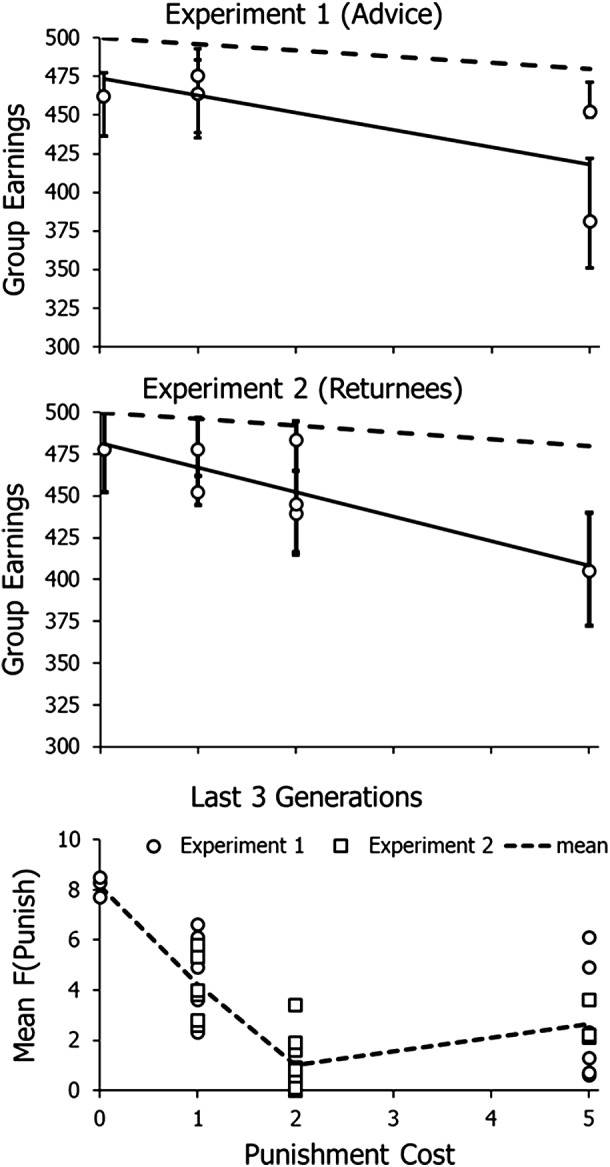
Overall functionality and punishing in Experiments 1 and 2. *Top*: group earnings in Experiment 1 (transmission by advice) as a function of punishment cost. Solid regression line is fitted to the circles, which represent median earnings. Error bars represent upper and lower quartiles. Dashed line represents maximum efficiency. *Middle*: group earnings in Experiment 2. Lines and points as in the top graph. *Bottom*: mean choosing to punish in Experiments 1 and 2 as a function of punishment cost. Dashed lines fitted to the means across both experiments.

Figure 8 shows results in the last three generations of each condition as a function of cost of punishing. The bottom graph shows the mean frequency of choosing to punish. The level of punishing was high when punishing was free (mean = 8.2), less when punishing cost 1 cent (mean = 4.2) and lower still when punishing cost 2 cents or 5 cents (mean = 1.0 and 2.6).

The mean (4.2) for one-cent punishment may reflect evolution of a consistent policy of punishing at the group level. If one person contributed zero while the others contributed 50, the defector would earn 90 cents and the others would net 40 cents, but if four people punished, then the defector would net only 50 cents, the same as if everyone contributed 50. If more than four chose to punish, defecting would become deleterious. The mean of punishing with 1-cent punishment was almost exactly at the level to render defecting fruitless. Because different players punished both within and across generations, this outcome must be ascribed to the group as a whole.

The ideal policy for the group would be for everyone to contribute the maximum (50 cents) and for four people to punish every round to deter defecting – that is, cooperation with policing. The top graph in Fig. 8 shows the median group earnings in Experiment 1 together with the interquartile range as a function of cost of punishing. The solid line fits the medians. The dashed line shows what the groups’ earnings would have been if they had followed the ideal policy:

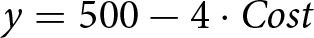


All points lie below this line, indicating that the groups failed to earn as much as would maximize net income. The middle graph in Fig. 8 shows the earnings in Experiment 2, and the results are much the same as in Experiment 1, with all points falling below the dashed line.

If we consider a group’s earnings as reflecting its functionality, say, in competition with other groups, then the top and middle graphs in Fig. 8 show that functionality decreased with the cost of punishing. That the solid line is steeper than the dashed line indicates faster decrease in functionality with punishment cost. Earnings depended on cost of punishing, number of players defecting and number of players punishing. When frequency of punishing was low, earnings depended heavily on number of players defecting, and, as expected, earnings varied more when punishing cost 2 or 5 cents, the conditions in which less punishing occurred.

One might have expected functionality with free punishment to be highest, but Fig. 8 shows this was not always so. In Experiment 1 (top graph), both 1-cent punishment conditions had higher earnings than in free punishment, and in Experiment 2, one 1-cent condition matched earnings with free punishment and one 2-cent punishment condition showed higher earnings than in free punishment. One reason is that the high level of free punishing was excessive. When someone defected, punishment of the defection was severe enough to impact the earnings of the group as a whole. This would be like ostracizing a member and then lacking that member in the event of a conflict with another group.

We found that using larger groups of 10 participants gave more room for evolution than smaller groups. Earlier work with groups of five indicated that a group often would consist entirely of extremely cooperative individuals. Larger groups ensured that we would almost always include some participants that would consider defecting. Even with 10 participants, we still occasionally recruited a group of super-cooperators, as indicated in Fig. 2.

We have shown here that we were able to repeat evolution with 1-cent cost of punishment four times, twice in Experiment 1 (Fig. 3) and then again with a different mode of transmission twice in Experiment 2 (Fig. 6). Also replicated in all four lineages was an initial decrease in choosing punishment, before punishment evolved, as if the lineage first established the need to punish before punishing (Fig. 2 middle and Fig. 5 top). We conclude that cultural evolution of cooperative altruistic punishing with low cost is probably a reliable, repeatable phenomenon. As with case studies and other studies with high cost per replication, science depends on accumulating evidence from many studies to overcome the low size of particular samples.

Because these experiments showed that low-cost punishing (but not free punishing) induces evolution of cooperative altruistic punishing, further research might focus particularly on that condition. Some of our procedural features were probably unnecessary. One could simplify the procedure by simply asking a participant to choose punishing just by making a mark, rather than doing subtraction, and by setting the criterion at 50, rather than requiring the participant to set a criterion. These simplifications would probably make choosing punishment easier, but likely would have little effect on evolution. If evolution of punishing is a reliable result, examining dynamics within and across generations might reveal mechanisms underlying evolution, as in Fig. 7. Additionally, one might study competition among groups by comparing earnings across multiple groups.

The generality of the present findings remains an open question. Would these results only be found in American college students, who come from a culture where ‘sharing is caring’ (Baum et al., 2012)? Replicating this experiment in a culture that is less cooperative than in the United States might produce different results (Herrmann et al., 2008). High rates of cooperation in many societies create a ceiling effect for the cumulative evolution of cooperation in the lab. In societies with less baseline cooperation, antisocial punishment values might retard the evolution of cooperation in the laboratory or, over a number of generations, perhaps people from such societies might overcome an initial tendency not to cooperate.

## 9. Conclusion

Computer simulations, field observations and experimental models all potentially throw light on cultural evolution. The present study offers a demonstration of a possible experimental model. We found similar cultural evolution of altruistic punishing across two different mechanisms of transmission across generations. Bringing back experienced elders may offer higher resemblance to the everyday world, but is logistically more difficult to implement. Altogether, these experiments point the way towards possible procedures for more close study of cultural evolution in the laboratory.

The strong effect of punishment cost in this study is interesting in the light of international variation in the costs imposed by states on citizens for punishment of political elites and on the costs citizens must pay to punish each other for non-cooperation. Liberal political regimes support free speech and easy and effective voting. In Sweden, Norway and Finland, where income tax records are public information, misbehaviour is relatively inexpensive to call out and sanction. More repressive regimes impose heavy costs on verbal dissent and make punishing misbehaviour costly. Less formally, subgroups such as stigmatized minorities may also be subject to heavy repressive sanctions from majority citizens as well as agents of the state, as in the Jim Crow era repression of Black citizens of the American South and pogroms often visited on the Jews of Europe. In the sample of countries studied by Herrmann et al. (2008), societies that could cooperate using prosocial punishment in the Public-Goods Game in the laboratory also had better national-level economic performance compared with those with high rates of antisocial punishment. The real-world impacts of punishment costs seem to be large.

## Research Transparency and Reproducibility

Data available from W.M.B. upon request.

## APPENDIX A. Instructions for Experiment 1

Below are the instructions for 5-cent punishment cost. The text in italics changed, depending on the cost.

### Instructions

**No talking with other participants is permitted. You may raise your hand to ask the researcher questions.**

This is a decision-making experiment that *involves no deception*. It is funded by research grants. At the beginning of the experiment, you are being given an endowment of $5.00 dollars. This is your money, and it appears on your record sheet as 500 cents in the last column labelled ‘Private Account’. Depending on the decisions made by you and others in the course of the experiment, this account could either grow or diminish. Whatever amount of money you end up with will become your personal property at the end of the experiment. **The money is yours to keep.**

The experiment runs for 10 rounds, and each will proceed in the same way. At the beginning of each round you must make a couple of decisions and at the end you may communicate a written message to the other participants.

### Procedures

Other participants will NOT be able know your personal decisions in this experiment. Because each person conducts decisions in the privacy of the cardboard box, and hands in the ‘record sheet’ and ‘communication folder’ every round, your anonymity will be preserved.

1. In each round, choose how many cents (ranging from 0 to 50) to allocate to the public account. For each round, the maximum contribution allowed is 50, but you may contribute anywhere from zero to 50 cents. At the end of each round, the researcher will sum all of the public account contributions, **double** this amount, and then distribute it equally among participants.
2. Look at your record sheet. Each round, you will write your contribution in the column labelled ‘Public Contribution’ (Column 2). In the next column (3), subtract your contribution from your private account (Column 9).
3. In the next column (4), indicate whether or not you want to punish anyone contributing too little to the public account. *Punishment costs 5 cents*. If you choose to punish, write a *five (5)* in Column 4 and subtract *five (5)* from Column 3 and write the result in Column 5. Then specify in Column 6 the contribution below which you want to levy a fine of 10 cents. Anyone who contributes less than the criterion will be fined. *You will not personally receive the punishment fines.* If you choose not to punish, leave Columns 4, 5 and 6 blank. The researchers will gather all the folders, make the appropriate calculations using the computer, announce the results, and write them on the whiteboard.
4. If you wish, use the ‘Communication Folder’ to write a brief message to the other participants. Write your message in the space allocated for each round. When complete, the researchers will gather the communication folders and read aloud word-for-word all of the messages. Remember, other participants will not be able to match your identity with your message. If you have no message, just write ‘no message’, so everybody writes something.
5. Unless you are in the first group to participate in this experiment, when you start the experiment you will receive advice on how to make your decisions from the group who participated in the experiment immediately prior to you. This advice will be read out loud. People find this situation a bit complicated, and you will do well to attend to this advice from the previous generation, who had 10 rounds of experience and also had the benefit of advice from generations before them. At the end of your 10 rounds, you will leave advice on how to make decisions for the next group.

### Subject # _____ Group # _______ Date _________________________

**Table:**

1	2	3	4	5	6	7	8	9
Round	Public Contribution (cents)	Private Acct. = Subtract Col. 2 from Col.9	Choose: Punish = 5 cents	Private Acct. = Subtract Col.4 from Col.3	Criterion for punishing	Share from Public Acct.	Subtract due to punish-ment	Private Acct. = (500 cents to Starts); Add Cols. 5,7, and 8
**1**								
**2**								
**3**								
**4**								
**5**								
**6**								
**7**								
**8**								
**9**								
**10**								

## APPENDIX B. Instructions for Experiment 2

Below are the instructions for 1-cent punishment cost. Text in italics changed, depending on cost.

### Instructions

**No talking with other participants is permitted. You may raise your hand to ask the researcher questions.**

This is a decision-making experiment that *involves no deception*. At the beginning of the experiment, you are being given an endowment of $5.00 dollars. This is your money, and it appears on your record sheet as 500 cents in the last column labelled ‘Private Account’. Depending on the decisions made by you and others in the course of the experiment, this account could either grow or diminish. Whatever amount of money you end up with will become your personal property at the end of the experiment. **The money is yours to keep.**

The experiment runs for 10 rounds, and each will proceed in the same way. At the beginning of each round you may communicate a written message to the other participants, and then you must make a couple of decisions.

### Procedures

Other participants will NOT be able know your personal decisions in this experiment. Because each person conducts decisions in the privacy of the cardboard box, and hands in the ‘communication folder’ and ‘record sheet’ every round, your anonymity will be preserved.

1. If you wish, use the ‘Communication Folder’ before each round to write a brief message to the other participants. Write your message in the space allocated for each round. When complete, the researchers will gather the communication folders and read aloud word-for-word all of the messages. Remember, other participants will not be able to match your identity with your message. If you have no message, just write ‘no message’, so everybody writes something.
2. In each group except for the first, some people will have participated in the experiment before. People find this situation a bit complicated, and you will do well to attend to the advice from the previous generation, who already had 10 rounds of experience.
3. After the messages have been read, choose how many cents (ranging from 0 to 50) to allocate to the public account. For each round, the maximum contribution allowed is 50, but you may contribute anywhere from zero to 50 cents. At the end of each round, the researcher will sum all of the public account contributions, **double** this amount, and then distribute it equally among participants.
4. Look at your record sheet. Each round, you will write your contribution in the column labelled ‘Public Contribution’ (Column 2). In the next column (3), subtract your contribution from whatever is in your private account (Column 9), beginning at 500, but changing from round to round.
5. In the next column (4), indicate whether or not you want to punish anyone contributing too little to the public account. *Punishment costs 1 cent*. If you choose to punish, write a *one (1)* in Column 4 and subtract *one (1)* from Column 3 and write the result in Column 5. Then specify in Column 6 the contribution (1 to 50) below which you want to levy a fine of 10 cents. Anyone who contributes less than the criterion will be fined. Punishments stack: the more people punish, the more the fine. *You will not personally receive the money from the fines; it is just taken away.* If you choose not to punish, leave Columns 4, 5 and 6 blank. The researchers will gather all the folders, make the appropriate calculations using the computer, announce the results, and write them on the whiteboard.
